# Downregulation of angiotensin type 1 receptor and nuclear factor-κB by sirtuin 1 contributes to renoprotection in unilateral ureteral obstruction

**DOI:** 10.1038/srep33705

**Published:** 2016-09-23

**Authors:** Shao-Yu Yang, Shuei-Liong Lin, Yung-Ming Chen, Vin-Cent Wu, Wei-Shiung Yang, Kwan-Dun Wu

**Affiliations:** 1 Graduate Institute of Clinical Medicine, National Taiwan University College of Medicine, Taipei, Taiwan; 2 Department of Internal Medicine, National Taiwan University Hospital and College of Medicine, Taipei, Taiwan; 3 Graduate Institute of Physiology, National Taiwan University College of Medicine, Taipei, Taiwan; 4 Department of Internal Medicine, National Taiwan University Hospital Yun-Lin Branch, Douliou City, Taiwan

## Abstract

Activation of sirtuin 1 (Sirt1) attenuates unilateral ureteral obstruction (UUO)-induced inflammation and fibrosis, suggesting that Sirt1 may prevent tubulointerstitial fibrosis. In this study, we explored changes in the expression of Sirt1 in the kidneys of UUO-treated rats and evaluated the effects of Sirt1 activation or inhibition on renal pathology and mediators of UUO pathogenesis, especially angiotensin II and nuclear factor (NF)-κB, in rats and rat renal fibroblasts. Sirt1 expression increased in the obstructed kidney but not in the contralateral kidney and was mainly detected in tubulointerstitial cells. Resveratrol, a Sirt1 activator, decreased UUO-induced inflammation and fibrosis, while sirtinol, a Sirt1 inhibitor, enhanced UUO-induced inflammation. UUO increased renal angiotensin type 1 receptor (AT1R), NF-κB, monocyte chemotactic protein 1 (MCP-1), and fibronectin expression. Resveratrol attenuated these UUO-induced changes, whereas sirtinol enhanced them, with the exception of fibronectin. In renal fibroblasts, Sirt1 overexpression reduced AT1R and NF-κB levels, while Sirt1 knockdown had the opposite effects. Sirtinol increased the levels of AT1R, NF-κB, MCP-1, and connective tissue growth factor (CTGF), while resveratrol reduced AT1R levels. Our results suggested that Sirt1 inhibited AT1R and NF-κB expression in renal fibroblasts and that these mechanisms may play roles in alleviating UUO-induced damages.

Sirtuin 1 (Sirt1), a nicotinamide adenine dinucleotide (NAD^+^)-dependent class III deacetylase that can deacetylate both histone and non-histone proteins and has been shown to participate in numerous cellular processes via deacetylation of specific substrates. In particular, Sirt1 has been shown to exert renoprotective effects in several models of acute kidney injury^1^,^2^,^3^ and chronic kidnefy diseases^4^,^5^.

Unilateral ureteral obstruction (UUO) is commonly used for exploring the pathogenesis of renal interstitial fibrosis, and several studies have shown that Sirt1 activators can improve UUO-induced apoptosis, renal interstitial inflammation, and fibrosis, whereas Sirt1 knockdown aggravates UUO-related apoptosis and fibrosis^6^,^7^,^8^. Although the detailed mechanisms of Sirt1-mediated renoprotection in UUO remain to be elucidated, these studies have suggested that Sirt1-dependent induction of cyclooxygenase-2^6^ and deacetylation of Smad3^7^ and STAT3^8^ may play a role. However, other factors associated with UUO pathogenesis may also be involved in mediating Sirt1-dependent renoprotection. Excessive activation of the local renin-angiotensin system, which leads to a prominent elevation of angiotensin II, has been linked to the progression of renal interstitial fibrosis and obstructive nephropathy^9^. Through binding to its receptor angiotensin type 1 receptor (AT1R), angiotensin II activates nuclear factor (NF)-κB and other downstream mediators, thereby inducing inflammation and fibrosis, which are thought to be directly related to the pathogenesis of UUO^10^. Given that Sirt1 consistently exhibits renoprotective properties in various kidney injuries and diseases, it is possible that Sirt1 attenuates activation of common signaling pathways involved in the progression of kidney pathology via interactions with upstream mediators, such as angiotensin II and NF-κB. In this study, we explored the effects of UUO and Sirt1 on important mediators in UUO pathogenesis. Furthermore, although a previous study showed that Sirt1 expression is significantly increased in the obstructed kidney^6^, this effect has not been localized to a particular part of or cell type in the affected kidney.

Therefore, in this study, we explored changes in Sirt1 expression and distribution in the obstructed kidney with the aim of elucidating the mechanisms underlying the renoprotective activity of Sirt1 in UUO.

## Results

### Increased Sirt1 expression in UUO kidneys

**Figure 1:**
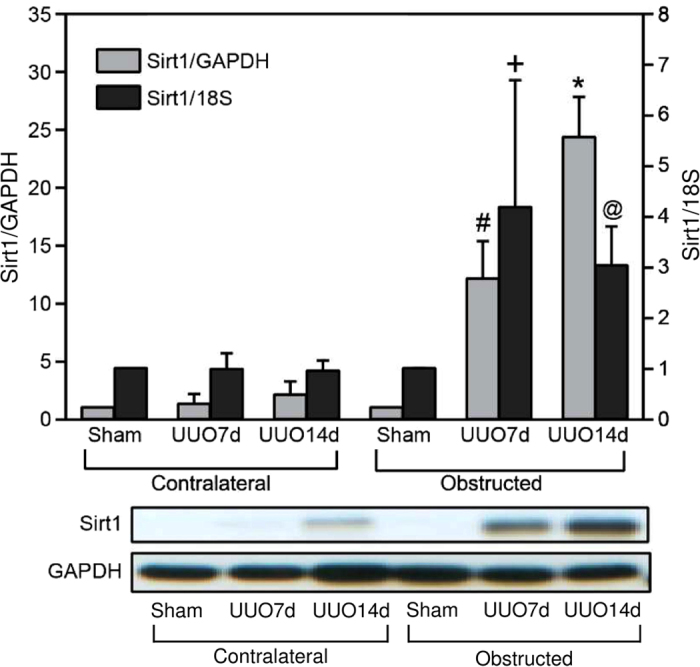
Sirt1 expression in the kidney increased after unilateral ureteral obstruction (UUO). Sirt1 protein and mRNA levels in the obstructed and contralateral kidneys were quantified by immunoblotting and real-time PCR, respectively, 7 (UUO7d) and 14 (UUO14d) days after UUO. A representative immunoblot is shown in the lower panel. The light grey bars are protein levels, and the Sirt1/GAPDH ratios are shown at left axis; the dark grey bars are mRNA levels, and the Sirt1/18S ratios are shown at right axis. ^+^*P* *=* 0.047, ^@^*P* = 0.005, ^#^*P* = 0.03, and *P = 0.007 versus sham operation (Sham). All gels were run under the same experimental conditions.

The expression of *Sirt1* mRNA in the obstructed kidneys was significantly increased on both days 7 and 14 after UUO compared with that in the kidneys of sham-operated rats; however, *Sirt1* expression in the contralateral kidney remained at the control level. Consistent with these increases in mRNA, Sirt1 protein was also elevated in the obstructed kidney (Fig. 1).

### UUO increased Sirt1 expression in tubuloepithelial cells, interstitial fibroblasts, and macrophages

**Figure 2:**
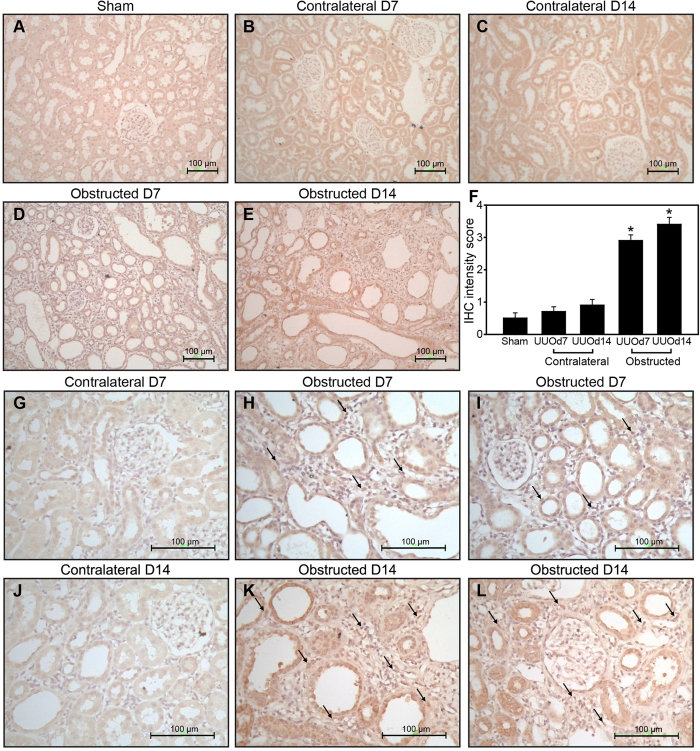
Immunohistochemistry (IHC) analysis of Sirt1 expression in rat kidneys after unilateral ureteral obstruction (UUO). IHC of Sirt1 in the kidneys of sham-operated rats (**A**) contralateral kidney on days 7 (**B**) and 14 (**C**) and obstructed kidney on days 7 (**D**) and 14 (**E**) after UUO are shown (magnification, 200×). Sirt1 IHC intensity scores are shown for the above samples (**F**) IHC of Sirt1 in the contralateral kidney (**G**) interstitium (**H**) and area near the glomeruli (**I**) of the obstructed kidney on day 7 after UUO. IHC of Sirt1 in the contralateral kidney (**J**), interstitium (**K**) and area near the glomeruli (**L**) of the obstructed kidney on day14 after UUO. The arrows indicate increased Sirt1 expression in some interstitial cells (magnification, 400×). *P < 0.001.

In sham-operated rats, Sirt1 was expressed weakly in some tubuloepithelial cells. In UUO-treated rats, the increase in Sirt1 expression was mainly observed in tubuloepithelial cells and the renal interstitium in the obstructed kidney. In contrast, no changes were detected in the contralateral kidneys (Fig. 2A–E). The Sirt1 IHC intensity score increased significantly in the obstructed kidneys after UUO, but remained unchanged in the contralateral kidneys (Fig. 2F). Comparing to the cortex, the Sirt1 expression in the medulla was more abundant in the sham-operated rat kidneys; in response to UUO, Sirt1 expression in the medulla remained more abundant than that in the cortex, both in the contralateral and obstructed kidneys (Supplementary Figure S1).

After UUO, increased Sirt1 expression was observed in many interstitial cells in the obstructed kidneys, but not in the contralateral kidneys. The morphology of the interstitial cells with increased Sirt1 mostly resembled that of fibroblasts or monocytes/macrophages (Fig. 2G–L, Supplementary Figure S2). Immunofluorescence revealed some faint co-localization of Sirt1 and ED-1 and some faint co-localization of Sirt1 and fibronectin in the renal interstitium (Supplementary Figure S3).

### Alterations in UUO-induced inflammation/fibrosis by resveratrol and sirtinol

After UUO, the ratio of kidney weight to body weight remained unchanged after UUO (0.55 ± 0.05 v.s. 0.60 ± 0.06%, *P *= 0.17). The treatment of resveratrol or sirtinol did not change the ratio of kidney weight to body weight after UUO (*P* = 0.35 and *P* = 0.11 respectively). The plasma creatinine levels did not change significantly after UUO or after the pharmacological intervention (Sham: 0.37 ± 0.03, UUO: 0.47 ± 0.03, UUO & resveratrol: 0.40 ± 0.06, UUO & sirtinol: 0.47 ± 0.03 mg/dL).

**Figure 3:**
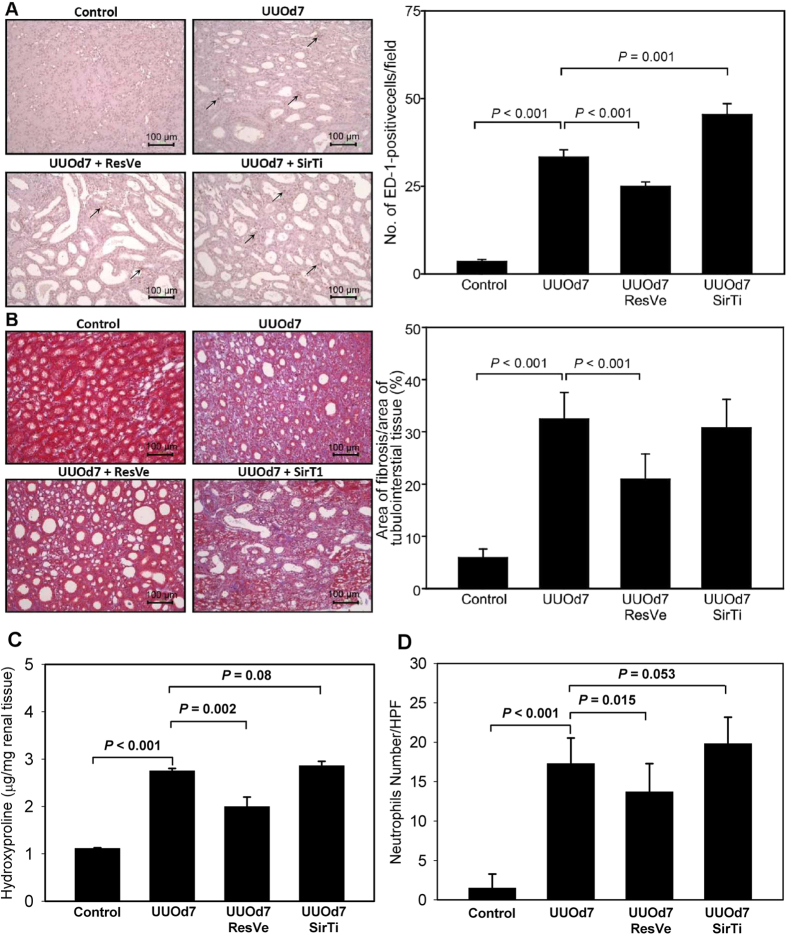
Alterations in inflammation and fibrosis markers after unilateral ureteral obstruction in response to Sirt1 activation or inhibition. (**A**) Immunohistochemistry for ED-1 (magnification, 200×) shows infiltration of ED-1-positive cells at 7 days after UUO (UUOd7). The number of ED-1-positive cells per field decreased after resveratrol (ResVe) treatment but increased after sirtinol (SirTi) treatment for 7 days. The arrows indicate some of the ED-1 positive cells. (**B**) Masson’s trichrome stain (magnification, 200×) shows fibrosis in the kidney at 7 days after UUO (UUOd7), and the ratio of the area of fibrosis decreased after resveratrol (ResVe) treatment but did not change significantly after sirtinol (SirTi) treatment for 7 days. (**C**) Hydroxyproline content determined from the entire kidney of control, obstructed kidneys 7 days after UUO (UUOd7), with resveratrol (ResVe) or sirtinol (SirTi) intervention for 7 days after UUO (n = 3 in each group). (**D**) Neutrophils infiltration count in the kidneys from above four groups (10 random HPFs in each group).

UUO increased inflammatory cells infiltration after 7 days. Activation of Sirt1 by resveratrol reduced the observed increase in inflammatory cell infiltration, whereas inhibition of Sirt1 by sirtinol resulted in greater increases in inflammatory cell infiltration (Fig. 3A). By analyzing the ratio of area of fibrosis with Masson’s trichrome stain, UUO increased fibrosis significantly after 7 days, and this effect was attenuated by resveratrol. Sirtinol did not significantly affect UUO-induced fibrosis (*P* = 0.196, Fig. 3B). UUO increased the level of hydroxyproline content, another marker of renal fibrosis (*P* < 0.001, Fig. 3C). Resveratrol ameliorated this UUO-induced alteration (*P* = 0.002), while sirtinol did not influence hydroxyproline content significantly (*P* = 0.08). Neutrophils infiltration increased 7 days after UUO (*P* < 0.001, Fig. 3D). The increased neutrophils infiltration was attenuated by resveratrol (*P* = 0.015) and had a trend to be worsened by sirtinol (*P* = 0.053).

### Alterations in UUO-induced apoptosis/oxidative stress by resveratrol and sirtinol

**Figure 4:**
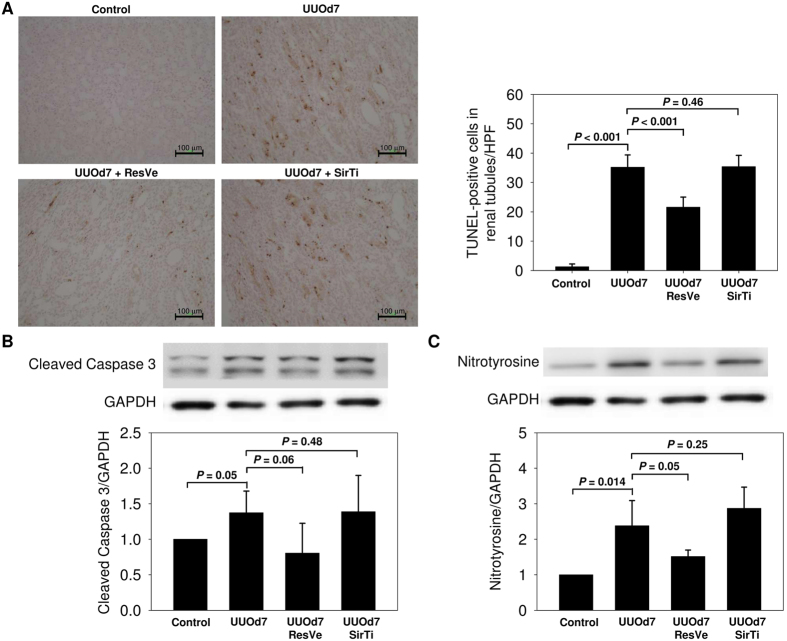
Alterations in apoptosis and oxidative stress markers after unilateral ureteral obstruction in response to Sirt1 activation or inhibition. (**A**) Representative pictures (original magnification, ×200) and quantification of TUNEL-positive apoptosis in the renal medulla of control, obstructed kidney 7 days after UUO (UUOd7), with resveratrol (ResVe) or sirtinol (SirTi) intervention for 7 days after UUO. (**B**) Immunoblot for the apoptosis marker cleaved caspase-3 in the entire kidney of above four groups (n = 3 in each group). (**C**) Immunoblot for the oxidative stress marker nitrotyrosine in the entire kidney of above four groups (n = 3 in each group).

By TUNEL assay, apoptosis was seen predominantly in the renal medulla of rats 7 days after UUO (Fig. 4A). TUNEL-positive apoptosis was significantly greater 7 days after UUO (*P* < 0.001), and UUO-induced apoptosis was attenuated significantly by resveratrol (*P* < 0.001). Sirtinol did not have obvious effect on UUO-induced apoptosis (*P* = 0.46). Increased apoptosis in the obstructed kidney after UUO was further confirmed by higher levels of cleaved caspase-3 expression comparing with the controls (*P* = 0.05, Fig. 4B). Resveratrol had a borderline trend to reduce UUO-induced increase of cleaved caspase-3 (*P* = 0.06), while sirtinol did not have significant effect on cleaved caspase-3 level (*P *= 0.48).

The level of renal nitrotyrosine, a marker of oxidative stress, increased significantly after UUO (*P* = 0.014, Fig. 4C), and the increased nitrotyrosine level was reversed partially by resveratrol (*P* = 0.05). Sirtinol did not have significant effect on nitrotyrosine level (*P* = 0.25).

### UUO-induced changes in renal pro-inflammatory mediators were attenuated by resveratrol but enhanced by sirtinol

**Figure 5:**
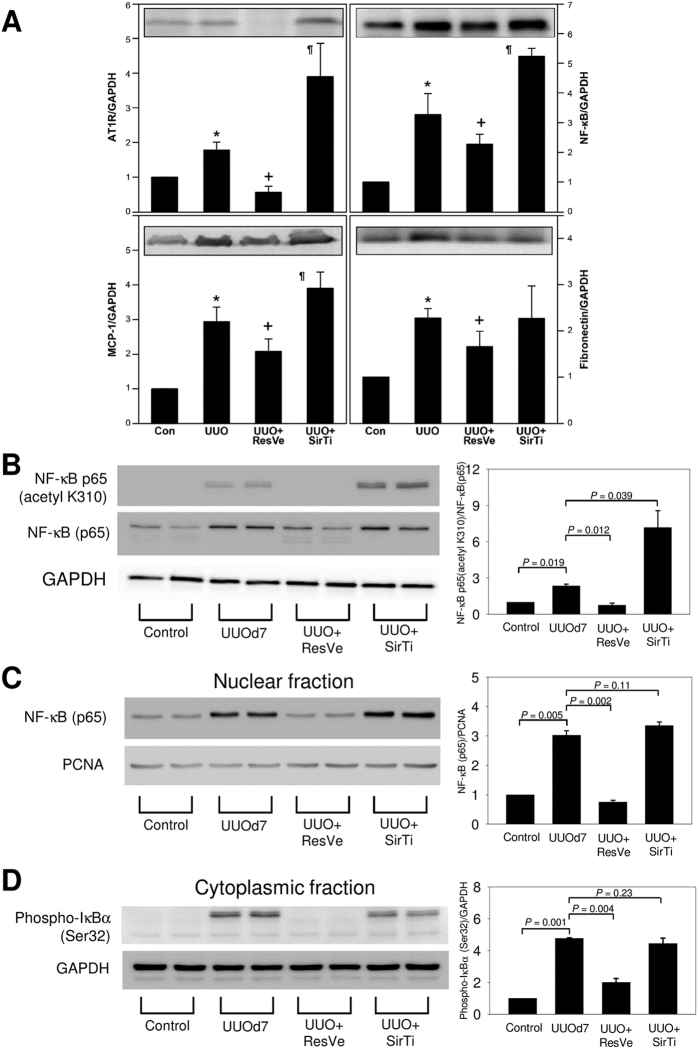
Changes in important mediators of inflammation and fibrosis in the obstructed kidney 7 days after UUO and the effects of Sirt1 activation or inhibition. (**A**) Representative immunoblot and quantification of angiotensin II type 1 receptor (AT1R), nuclear factor-κB (NF-κB), monocyte chemotactic protein 1 (MCP-1), and fibronectin in the kidneys of the controls, obstructed kidneys at 7 days after UUO (UUO), and obstructed kidneys with resveratrol (ResVe) or sirtinol (SirTi) intervention for 7 days after UUO. *P < 0.05 versus the control group, ^+^*P* < 0.05 versus the UUO group, ^¶^*P* < 0.05 versus the UUO group. (**B**) Representative immunoblot and quantification of NF-κB p65 (acetyl K310), NF-κB (p65) and GAPDH. (**C**) Representative immunoblot and quantification of NF-κB (p65) and proliferating cell nuclear antigen (PCNA) in the nuclear fraction. (**D**) Representative immunoblot and quantification of phospho-IκBα (Ser32) and GAPDH in the cytoplasmic fraction.

The expression levels of AT1R, NF-κB, MCP-1, and fibronectin increased significantly 7 days after UUO. Resveratrol treatment for 7 days attenuated these changes, while sirtinol treatment enhanced these changes, except for the change in fibronectin expression (Fig. 5A). The ratio of acetyl (K310)-NF-κB (p65) to NF-κB (p65) increased significantly after UUO. Resveratrol treatment ameliorated this UUO-induced NF-κB acetylation, while sirtinol aggravated it (Fig. 5B). Both nuclear NF-κB (p65) and cytoplasmic phospho-IκBα levels increased after UUO, and resveratrol treatment ameliorated these UUO-induced NF-κB activation changes (Fig. 5C,D). The angiotensin II type 2 receptor (AT2R) expression did not change significantly after UUO or Sirt1 activation/inhibition (Supplementary Figure S4).

### Inhibition of AT1R expression by Sirt1 in NRK-49F cells

**Figure 6:**
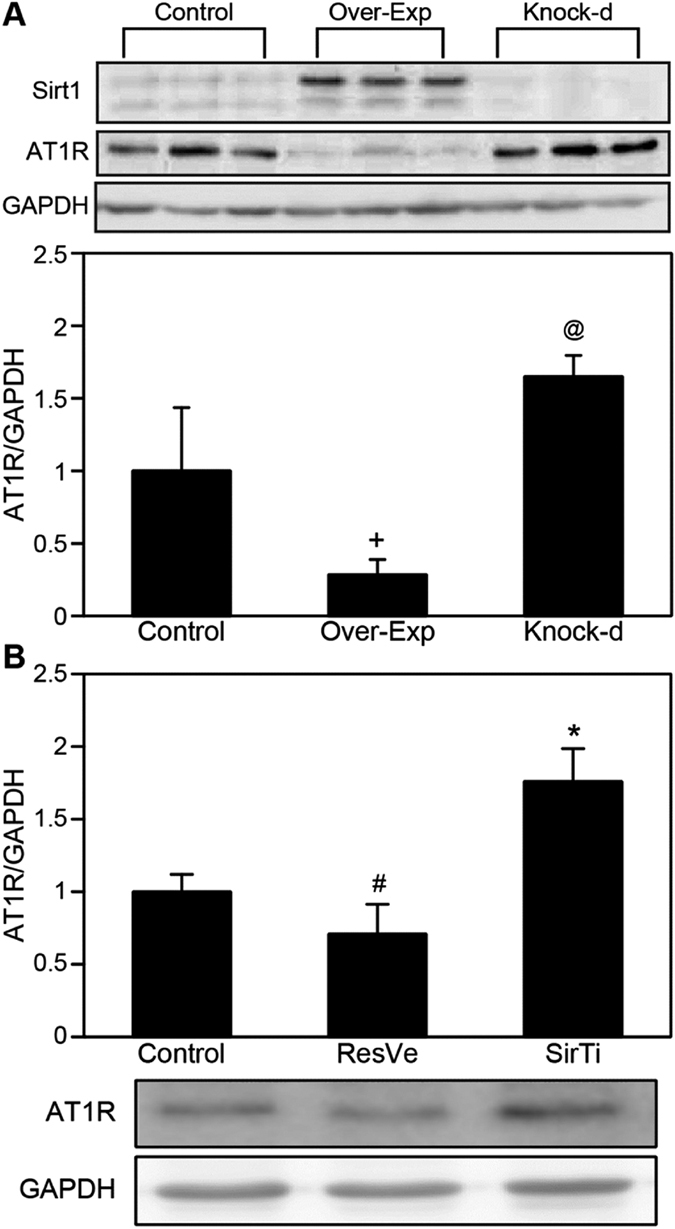
Sirt1 inhibited angiotensin II type 1 receptor (AT1R) expression in NRK-49F cells. (**A**) Representative immunoblot and quantification of AT1R expression in control, Sirt1-overexpressing (Over-Exp), and Sirt1-knockdown (Knock-d) NRK-49F cells. (**B**) Representative immunoblot and quantification of AT1R expression after treatment with 50 μM resveratrol (ResVe) or sirtinol (SirTi) for 24 h. ^+^*P* = 0.026, ^@^*P* = 0.035, ^#^*P* = 0.050, and *P = 0.003 versus the control group. Cropped blots are shown, and all the gels were run under the same experimental conditions.

The effect of Sirt1 was examined by modulating its expression in NRK-49F cells. AT1R expression was significantly upregulated after Sirt1 knockdown, but downregulated in Sirt1-overexpressing cells (Fig. 6A). A decrease in AT1R expression in NRK-49F cells was observed after the addition of the Sirt1 activator resveratrol at 50 μM for 24 h (*P* = 0.0498), whereas treatment with the Sirt1 inhibitor sirtinol at 50 μM for 24 h significantly increased AT1R levels in NRK-49F cells (*P* = 0.0034; Fig. 6B).

### Inhibitory effects of Sirt1 on NF-κB expression

**Figure 7:**
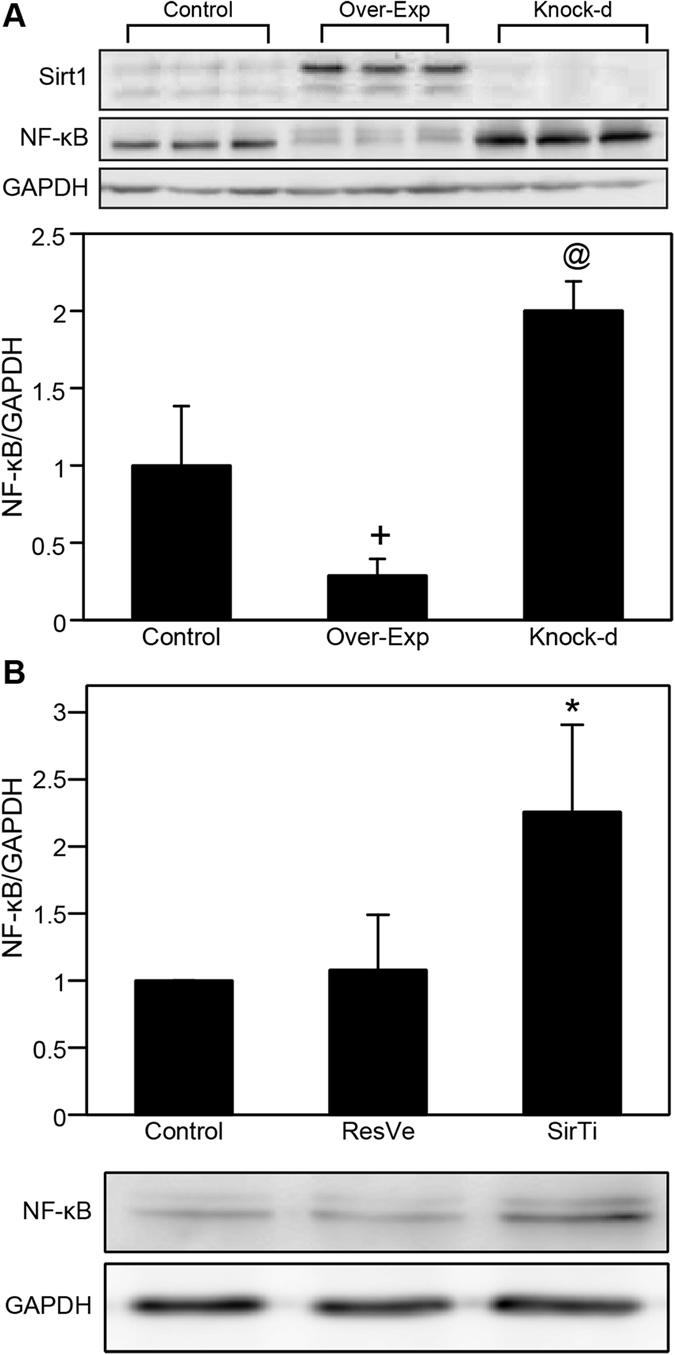
Sirt1 inhibits nuclear factor-κB (NF-κB) expression in NRK-49F cells. (**A**) Representative immunoblot of Sirt1 and NF-κB expression in control, Sirt1-overexpressing (Over-Exp), and Sirt1-knockdown (Knock-d) NRK-49F cells. (**B**) Representative immunoblot and quantification of NF-κB expression after treatment with 50 μM resveratrol (ResVe) or sirtinol (SirTi) for 24 h. ^+^*P* = 0.018, ^@^*P* = 0.008, *P = 0.015 versus the control group. Cropped blots are shown, and all gels were run under the same experimental conditions.

Sirt1 overexpression decreased NF-κB expression in NRK-49F cells; however, Sirt1 knockdown had the opposite effect (Fig. 7A). Treatment with 50 μM sirtinol for 24 h significantly increased NF-κB; however, the addition of 50 μM resveratrol did not induce any changes (Fig. 7B).

### Effects of Sirt1 on the expression of factors related to inflammation and fibrosis

**Figure 8:**
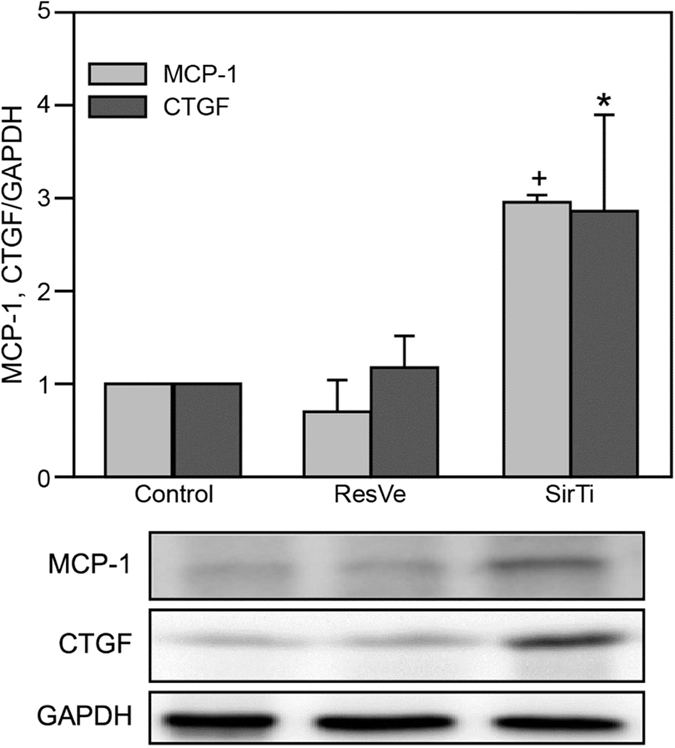
Sirt1 inhibited the expression of MCP-1 and CTGF in NRK-49F cells. Representative immunoblot and quantification of MCP-1 and CTGF expression in NRK-49F cells after treatment with 50 μM resveratrol (ResVe) or sirtinol (SirTi) for 24 h. ^+^*P* = 0.0002 and *P = 0.031 versus the control group. Cropped blots are shown, and all gels were run under the same experimental conditions.

The expression levels of both MCP-1 and CTGF were significantly increased in NRK-49F cells treated with 50 μM sirtinol for 24 h. in contrast, treatment with 50 μM resveratrol did not cause significant changes in the expression levels of these proteins (Fig. 8).

## Discussion

Sirt1 has been shown to exert renoprotective effects in *in vivo* models of UUO, and several underlying mechanisms have been proposed^6^,^7^,^8^. However, the cells responsible for the increase in Sirt1 expression in the obstructed kidneys have not been identified. In the present study, we showed that Sirt1 expression was markedly elevated in tubuloepithelial and interstitial cells in the UUO-treated kidney. We also found that *in vivo* UUO-induced inflammation and fibrosis could be attenuated by resveratrol, a Sirt1 activator, while UUO-induced inflammation was enhanced by sirtinol, a Sirt1 inhibitor. UUO-induced upregulation of AT1R, NF-κB, MCP-1, and fibronectin was attenuated by resveratrol but increased by sirtinol, with the exception of fibronectin. The activation and proliferation of fibroblasts play important roles in the pathogenesis of UUO-induced fibrosis of the kidney^11^; therefore, we evaluated the effects of Sirt1 on mediators of UUO pathogenesis in renal interstitial fibroblasts. Our results showed that overexpression of Sirt1 downregulated the expression of AT1R and NF-κB, whereas knockdown of Sirt1 had the opposite effects. *In vitro* sirtinol upregulated the expression of AT1R, NF-κB, MCP-1, and CTGF in renal interstitial fibroblasts, while resveratrol downregulated the expression of AT1R. Thus, these results provided important insights into the mechanisms through which Sirt1 regulates inflammation and fibrosis in the kidneys.

The mechanisms mediating increased Sirt1 expression in the obstructed kidneys of UUO rats are not fully understood. NF-κB activation has been shown to upregulate Sirt1 expression^12^. Thus, the angiotensin II-induced activation and expression of NF-κB in UUO may lead to Sirt1 upregulation in the kidney. Sirt1 upregulation may help cells to resist stresses and prevent UUO-induced apoptosis, inflammation, and fibrosis, as shown in previous studies^6^,^7^,^8^. However, Sirt1 upregulation alone is not sufficient for mitigating the effects of all UUO-induced damage. The combination of upregulation and activation of Sirt1 has been shown to be more effective in attenuating UUO-induced damage, as shown by the effects of Sirt1 activators^6^,^7^,^8^. This notion is supported by previous studies showing that heterozygous Sirt1-knockout mice exhibit a less obvious increase in Sirt1 expression after UUO, concomitant with increased UUO-induced damage^6^.

UUO-induced changes *in vivo*, including increased AT1R, NF-κB, MCP-1, and fibronectin expression, were attenuated by resveratrol treatment for 7 days and enhanced by sirtinol treatment, except fibronectin; these results were consistent with the finding that fibrosis was not altered by sirtinol in Masson’s trichrome staining and hydroxyproline assay. However, it is unclear why sirtinol, a Sirt1 inhibitor, does not exacerbate fibrosis in the obstructed kidney after UUO. One possible explanation is that UUO is already a potent fibrogenic factor, so the effects of sirtinol on fibrosis and fibronectin expression may be masked by UUO. This hypothesis was supported by previous study showing that heterozygous Sirt1-knockout mice exhibit increased fibrosis and collagen I expression after UUO^6^, indicating that specific Sirt1 deficiency does worsen fibrosis after UUO. However, in a previous study, conflicting results were obtained^13^, showing that administration of sirtinol 50 mg/kg daily for 6 days in a mouse model of UUO attenuated collagen fibril deposition and reduced the expression of α-smooth muscle actin, collagen I, and fibronectin in the obstructed kidney. The discrepancies between this study and ours may be associated with the dose of sirtinol (50 mg/kg versus 2.5 mg/kg daily) and the model animals used (C57 mice versus Wistar rats). With the dose used in our study, sirtinol enhanced UUO-induced inflammatory cell infiltration and upregulation of AT1R, NF-κB, and MCP-1. However, higher dose of sirtinol may have other pharmacological effects in addition to Sirt1 inhibition, such as Sirt2 inhibition^13^, and such nonspecific pharmacological effects may interfere with the interpretation of Sirt1 inhibition *in vivo*. While analysis of heterozygous Sirt1-knockout mice supported that Sirt1 deficiency enhanced UUO-induced apoptosis and fibrosis, our study also demonstrated that sirtinol, probably via Sirt1 inhibition, enhanced UUO-induced inflammation.

UUO-induced inflammation and fibrosis are attenuated in AT1R-deficient mice^14^. Therefore, the renoprotective effects of Sirt1 activation in UUO rats may, at least in part, be exerted via suppression of AT1R expression. We found that UUO-induced increases in AT1R levels were significantly inhibited by treatment with resveratrol, decreasing to less than that observed in the control group. This observation may be explained by the combination of markedly increased Sirt1 expression after UUO and the activation of Sirt1 by resveratrol. *In vitro*, we also found that overexpression of Sirt1 in 49F cells reduced AT1R expression to 29% that in the control group. In vascular smooth muscle cells, Miyazaki *et al*. demonstrated that Sirt1 activation reduces AT1R expression, and showed that resveratrol inhibits Sp1 binding to AT1R gene promoter, and reduces AT1R gene transcription^15^. However, the mechanism of Sirt1-induced AT1R down-regulation in the kidney remains unclear and requires further study.

NF-κB is an important inflammatory mediator downstream of angiotensin II. Our study showed that Sirt1 overexpression in renal fibroblasts reduced NF-κB levels, potentially occurring downstream of the decreased AT1R expression described above. Previous studies^16^,^17^ have shown that Sirt1 inhibits NF-κB activity by deacetylating lysine 310. Our results also showed that resveratrol ameliorated UUO-induced NF-κB lysine 310 acetylation, while sirtinol aggravated it. Therefore, the use of resveratrol in UUO may attenuate inflammation by inhibiting both NF-κB expression and activation, and then inhibiting the transcriptional activity of downstream inflammatory mediators.

MCP-1 and CTGF, other important downstream mediators of renal tubulointerstitial inflammation and fibrosis in UUO, have been shown to be significantly increased in the obstructed kidney^9^,^18^. Renal interstitial fibroblasts express MCP-1 and may play an active role in the recruitment of inflammatory leucocytes into the interstitium^19^. Our finding that Sirt1 inhibition increased the levels of MCP-1 and CTGF supports the notion that the renoprotective effect of Sirt1 is related to the inhibition of inflammation and fibrosis in the interstitial fibroblasts.

Interestingly, resveratrol did not decrease NF-κB, MCP-1, and CTGF expression, possibly because baseline NF-κB, MCP-1, and CTGF levels were not high in fibroblasts, preventing further inhibition by resveratrol. However, the fibroblasts are activated after UUO, and the elevated NF-κB, MCP-1, and fibronectin do be reduced by resveratrol *in vivo*. In addition to fibroblasts, increased Sirt1 expression was also identified in interstitial macrophages/monocytes and tubuloepithelial cells in our study. Although we did not explore the biological significance of elevated Sirt1 expression in these cells, previous studies have described the roles of these cells in kidney fibrosis. For example, bone marrow monocytes are recruited to the obstructed kidney and differentiate into profibrotic kidney macrophages^20^, and the ablation of Sirt1 in macrophages renders NF-κB hyperacetylated, resulting in increased transcriptional activation of pro-inflammatory genes^21^. Accordingly, Sirt1 may play an important role in inhibiting macrophage-mediated inflammation in the obstructed kidney. Additionally, the Sirt1 activator resveratrol has been shown to block angiotensin II-induced expression of fibrotic genes in rat renal tubular epithelial NRK-52E cells^8^. Although fibrotic gene expression in tubuloepithelial cells is considered a marker of the epithelial to mesenchymal transition (EMT), the role of the EMT in UUO-induced renal interstitial fibrosis remains questionable^22^.

There are some limitations to this study. First, Sirt1 may simultaneously influence several mediators of UUO-induced oxidative stress, inflammation and fibrosis-related signaling, and their interaction may complicate the analysis of the overall effects of Sirt1 on these processes. Our study demonstrated that Sirt1 inhibits renal fibrosis at least partially by inhibiting AT1R and NF-κB expression, and previous studies also supported that Sirt1 inhibits renal fibrosis by inducing COX2, deacetylating Smad3 and STAT3. Second, UUO is a complex process that is likely to involve several types of kidney cells, and the effects of Sirt1 on cells subjected to UUO-induced damage remain to be elucidated.

In summary, the renoprotective effects of Sirt1 in UUO rats may result from inhibition of the angiotensin-NF-κB pathway. Our study provides a basis for the development of an alternative therapeutic option for renal interstitial fibrosis other than current options involving the use of angiotensin-converting enzyme inhibitors and AT1R blockers. Furthermore, Sirt1 activators may provide synergic effects when used in combination with the renin-angiotensin system blockade.

## Materials and Methods

### Animals

All experiments were performed in accordance with the protocols approved by the National Taiwan University College of Medicine and College of Public Health Institutional Animal Care and Use Committee and the Guide for the Care and Use of Laboratory Animals (Chinese-Taipei Society of Laboratory Animal Science). Male Wistar rats weighting 200–220 g each were obtained from the BioLASCO (Taiwan). The animals were housed under a 12-h light/dark cycle at the Laboratory Animal Center at the National Taiwan University College of Medicine and were allowed *ad libitum* access to water and food.

To study the effects of UUO on Sirt1 expression, UUO was modeled by ligating the left ureter; a sham operation was performed for the control animals. The rats were sacrificed on days 7 or 14 after the operation, and the kidneys were divided into three coronal sections and used for immunohistochemistry and RNA and protein extraction.

To explore the effects and mechanisms of pharmacological interventions of Sirt1 after UUO, the rats were divided into four groups: control (received sham operation), UUO (received UUO operation), UUO + resveratrol (received intraperitoneal resveratrol, a Sirt1 activator [Sigma, St. Louis, MO, USA], 5 mg/kg in dimethylsulfoxide [DMSO; Sigma] daily after UUO for 7 days), and UUO + sirtinol (received intraperitoneal sirtinol, a Sirt1 inhibitor [Sigma], 2.5 mg/kg in DMSO daily after UUO for 7 days). The doses of resveratrol and sirtinol used in our experiments were based on previous studies^7^,^23^. The rats in the control and UUO groups also received the same volume of DMSO daily by intraperitoneal injection. The rats were sacrificed on day 7 after the operation, and the kidneys were divided into three coronal sections and used for renal pathology and immunoblotting. Three rats were used in each group for comparison.

### Cell culture, Sirt1 overexpression and knockdown, and cell treatment

Normal rat kidney fibroblasts (NRK-49F cells) were cultured as described previously^24^. Briefly, cells were maintained in Dulbecco’s modified Eagle’s medium (Mediatech, Manassas, VA, USA) and supplemented with 10% fetal bovine serum (Biological Industries, Beit-Haemek, Israel) at 37 °C in a humidified 5% CO_2_ incubator. For Sirt1 knockdown, the cells were transduced with lentiviral particles carrying Sirt1 shRNA, according to the manufacturer’s instructions (Santa Cruz Biotechnology, Dallas, TX, USA). Briefly, culture medium was replaced with 2 mL of complete medium containing 5 μg/mL polybrene (Santa Cruz); then, 3 μL of lentiviral particles (2.5 × 10^4^ infectious units) was added, and cell monolayers were incubated for 24 h. Lentivirus-infected cells were selected by puromycin (Sigma) for 7 days, and the efficiency of Sirt1 knockdown was analyzed by reverse transcription polymerase chain reaction (RT-PCR) and immunoblotting. For overexpression of Sirt1, 2 μg of the pCruzHA SIRT1 plasmid carrying the *SIRT1* gene (Addgene, Cambridge, MA, USA) was diluted in 100 μL of serum-free medium containing 4 μL TurboFect reagent (Fermentas, Glen Burnie, MD, USA). The mixture was incubated for 20 min at room temperature and then added drop-wise to the medium. Four hours later, the culture medium was replaced with 2 mL complete medium. After 48 h, 400 mg/L G418 (Bionovas, Ann Arbor, MI, USA) was added to select Sirt1-overexpressing clones; the concentration was then changed to 200 mg/L for maintenance. After 10 days, Sirt1 expression was examined by RT-PCR and immunoblotting. Additionally, to evaluate the effects of pharmacological interventions, the cells without overexpression or knockdown were also treated with resveratrol or sirtinol at a concentration of 50 μM for 24 h, and the expression of target molecules was analyzed by immunoblotting. At least three separate cell seedings in successive days to weeks were used for dependability.

### RNA extraction and quantitative real-time PCR

Total RNA was extracted from homogenized kidneys or cultured cells with TRIzol reagent (Invitrogen, Carlsbad, CA, USA), and 10 μg RNA was reverse-transcribed using a High-Capacity cDNA Reverse Transcription Kit (Applied Biosystems, Foster City, CA, USA) according to the manufacturer’s instructions. Quantitative real-time PCR was performed using SYBR Green detection and an iQ5 instrument (Bio-Rad, Hercules, CA, USA). The cycling conditions were as follows: 95 °C for 10 min, followed by 40 cycles of 95 °C for 10 s, and 60 °C for 30 s. Melting curve analysis was performed, and threshold values were determined after normalization to those of 18S ribosomal RNA using Bio-Rad iQ5 Software 2.0. Data are presented as the fold induction relative to the control. The primers used in this study were as follows: *SIRT1* forward, 5′-CAGTGTCATGGTTCCTTTGC-3′ and *SIRT1* reverse, 5′-CACCGAGGAACTACCTGAT-3′; and 18S forward, 5′-AGTCCCTGCCCTTTGTACACA -3′ and 18S reverse 5′-CGATCCGAGGGCCTCACTA-3′. The target genes were amplified using GoTaq Green Master mix (Promega, Madison, WI, USA) according to the manufacturer’s protocol. PCR conditions were set as follows: initial template denaturation at 95 °C for 3 min, followed by 25 cycles of denaturation at 95 °C for 30 s, primer annealing at 60 °C for 30 s, and elongation at 72 °C for 45 s.

### Immunoblotting

Total proteins were extracted from cultured cells or homogenized renal tissues using radioimmunoprecipitation assay (RIPA) buffer (Sigma), and western blotting analysis was performed as described previously^18^. The following primary antibodies were used: rabbit polyclonal anti-Sirt1 (1:800; Sigma), anti-cleaved caspase-3 (1:1000; Cell Signaling Technology, Danvers, MA), anti-nitrotyrosine (1:1000; Santa Cruz), anti-AT1R (1:500; Millipore), anti-AT2R (1:500; Millipore), anti-NF-κB (p65) (1:5000; Enzo Life Science, Farmingdale, NY, USA), anti-acetyl (K310)-NF-kB p65 (1:1000; Abcam, Cambridge, MA), anti-phospho (Ser32)-IκBα (1:1000; Cell Signaling), anti-connective tissue growth factor (CTGF; 1:1000; Peprotech, Rocky Hill, NJ, USA), hamster monoclonal anti-monocyte chemotactic protein 1 (MCP-1; 1:500; Abcam), mouse monoclonal anti-fibronectin (1:400; Abcam), anti-GAPDH (1:2000; GenScript, Piscataway, NJ, USA), and anti-proliferating cell nuclear antigen (PCNA, 1:1000; GeneTex Inc., Irvine, CA). The secondary horseradish peroxidase (HRP)-conjugated antibodies included goat anti-rabbit IgG (Santa Cruz Biotechnology), goat anti-mouse IgG (PerkinElmer, Waltham, MA, USA), and goat anti-hamster IgG (Invitrogen) diluted 1:5000. Specific signals were visualized using Immobilon Western Chemiluminescent HRP Substrate (Millipore).

### Separation of the nuclear and cytoplasmic proteins

To separate the nuclear and cytoplasmic fractions for analysis of NF-κB pathways, a ProteoJET Cytoplasmic and Nuclear Protein Extraction Kit (Fermentas, Glen Burnie, MD) was used according to the manufacturer’s instruction. Briefly, the kidney tissue was homogenized, mixed with cell lysis buffer, and set on ice for 10 min. The cytoplasmic protein fraction was separated by centrifugation at 500 ×  *g* for 7 min at 4 °C, removing the supernatant to a new tube, centrifugation again at 20000 ×  *g* for 15 min at 4 °C, transferring the supernatant to a new tube again and stored at −70 °C before use. In addition, after washing the nuclei pellet with Nuclei washing buffer and discarding the supernatant, we added cold Nuclei storage buffer to the nuclei pellet. Next, we pipetted up and down, added nuclear lysis reagent, and mixed the solution via shaking for 15 min at 4 °C. We obtained the nuclear protein fraction by performing centrifugation at 20000 ×  *g* for 5 min at 4 °C, and then transferring the supernatant to a new tube, which was stored at −70 °C before use.

### Immunohistochemistry, Masson’s trichrome stain, and neutrophils infiltration count

Immunohistochemical analysis of renal tissues was performed as previously described^21^, using an Immunohistochemistry Accessory Kit (Bethyl Laboratories, Inc., Montgomery, TX, USA) according to the manufacturer’s recommendations. Briefly, after antigen retrieval with Epitope Retrieval Buffer, endogenous peroxidase activity was blocked with peroxidase quenching solution, and the sections were incubated with IHC Blocking Reagent (Bethyl) for 15 min at room temperature, followed by incubation with the primary anti-Sirt1 antibody (1:50; Santa Cruz Biotechnology) or anti-ED1 antibody (1:100, mouse monoclonal; Abcam) overnight at 4 °C. Then secondary antibody for IHC (Bethyl) was added, and sections were incubated for 1 h at room temperature, followed by washing and incubation with 3, 3′-diaminobenzidine chromogen. After counter-staining with hematoxylin (Sigma), the sections were covered with DPX mounting medium (Scharlau, Port Adelaide, Australia) and examined and photographed under a microscope equipped with a digital camera (Eclipse E400 with a DS-Fi1; Nikon, Japan).

For evaluating Sirt1 expression by IHC, 10 different fields were randomly selected and evaluated from each specimen, and a Sirt1 IHC intensity score was assigned semiquantitatively according to the percentage of the Sirt1-positive area (0: <5%, 1: 5–25%, 2: 25–50%, 3: 50–75%, 4: >75%). IHC of ED-1 was performed to evaluate the infiltration of inflammatory cells, and the number of ED-1-positive cells per field was counted in 15 randomly selected fields from each specimen. Masson’s trichrome staining was also performed to evaluate the collagen deposition in the kidney, and 15 tubulointerstitial fields from each specimen were analyzed. The area of collagen deposition was analyzed by measuring the percentage of the area of blue staining using threshold analysis with ImageJ v1.48 (National Institutes of Health, USA). The above analyses were performed at a magnification of 200× and were carried out by an investigator who was unaware of the origin of the slides. The neutrophils infiltration was counted on H&E-stained sections in 10 random HPFs at a magnification of 400×.

### Hydroxyproline assay

We used Hydroxyproline Assay Kit (Sigma) as manufacturer’s instructions. Briefly, each sample was prepared by 10 mg tissue homogenized in 100 μL of water, mixed with 100 μL of 12 M hydrochloric acid, and hydrolyzed at 120 °C for 3 h. After mixing with 5 mg of activated charcoal, the samples were centrifuged at 13,000 g for 2 min. The supernatant was transferred to a 96 well plate, and dried in a 60 °C oven. After adding Chloramine T/Oxidation Buffer Mixture (Sigma), the samples were incubated at room temperature for 5 min. After adding 100 μL of the diluted 4-(Dimethylamino)benzaldehyde Reagent, the samples were incubated for 90 min at 60 °C. The absorbance at 560 nm (A560) of the samples were measured with a spectrophotometric multiwell plate reader and the concentrations of hydroxyproline in the samples were determined by the standard curve set up in a same assay.

### TUNEL Assay

We used ApopTag Peroxidase *In Situ* Apoptosis Detection Kit (Millipore, Temecula, CA) as manufacturer’s instructions. Briefly, 5-μm-thick sections of paraffin-embedded tissue were dewaxed and hydrated, quenched in 3% H_2_O_2_ for 5 min to remove endogenous peroxidase activity, and then subjected to proteinase K treatment to expose DNA. After incubation in the Equilibration buffer (Millipore) for 1 min, the slides were incubated in terminal deoxynucleotidyl transferase (TdT) enzyme at 37 °C for 1 h in a humidified chamber. The reaction was terminated by putting the specimen in a coplin jar containing Stop/Wash buffer (Millipore), agitating for 15 s, and incubating for 10 min at room temperature. After washing with PBS for 3 times, the slides were incubated in anti-digoxigenin peroxidase (Millipore) at room temperature for 30 min and then stained with DAB. Quantification of apoptotic cells was performed by counting TUNEL-positive cells in 10 random HPFs (400×) in the renal medulla of one kidney section.

### Statistical analysis

Statistical analysis was performed using SPSS 20 software (SPSS Inc., Chicago, IL, USA). All data are expressed as the mean ± standard error. Statistical evaluation was performed by unpaired Student’s *t* test for two sets of data and analysis of variance (ANOVA) with a Bonferroni post-hoc test for multiple groups. Differences with *P* values of less than 0.05 were considered statistically significant.

## Additional Information

**How to cite this article**: Yang, S.-Y. *et al*. Downregulation of angiotensin type 1 receptor and nuclear factor-κB by sirtuin 1 contributes to renoprotection in unilateral ureteral obstruction. *Sci. Rep.* **6**, 33705; doi: 10.1038/srep33705 (2016).

## Supplementary Material

Supplementary Information
