# Supplementary material for: Downregulation of angiotensin type 1 receptor and nuclear factor-κB by sirtuin 1 contributes to renoprotection in unilateral ureteral obstruction
Source: Sci Rep. 2016 Sep 23;6:33705. doi: 10.1038/srep33705 (PMC5034227; doi:10.1038/srep33705)
Supplement: Supplementary Information [file srep33705-s1.pdf]

Supplementary Information

Downregulation of angiotensin type 1 receptor and nuclear factor-κB by sirtuin 1 contributes to renoprotection in unilateral ureteral obstruction

Shao-Yu Yang[1,2], Shuei-Liong Lin[2,3], Yung-Ming Chen[2,4], Vin-Cent Wu[2], Wei-Shiung Yang[1,2], Kwan-Dun Wu[2]*

[1]Graduate Institute of Clinical Medicine, National Taiwan University College of Medicine, Taipei, Taiwan; [2]Department of Internal Medicine, National Taiwan University Hospital and College of Medicine, Taipei, Taiwan; [3]Graduate Institute of Physiology, National Taiwan University College of Medicine, Taipei, Taiwan; [4]Department of Internal Medicine, National Taiwan University Hospital Yun-Lin Branch, Douliou City, Taiwan

*** Corresponding Author:**

Kwan-Dun Wu, MD, PhD

Department of Internal Medicine, National Taiwan University Hospital,

7 Chung-Shan South Road, Taipei 100, Taiwan

E-mail: kdwu@ntuh.gov.tw

MATERIALS AND METHODS

Immunofluorescence

The sections were incubated with 10% serum for 30 min at room temperature, and then with primary antibody at 4°C overnight. The primary antibodies included rabbit anti-Sirt1 (1:50; Sigma-Aldrich), mouse monoclonal anti-ED-1 (1:100; Abcam), and mouse monoclonal anti-fibronectin (1:100; Abcam) antibodies. After washing with phosphate-buffered saline (PBS), the sections were incubated with secondary antibody for 1 h at room temperature. The secondary antibodies included Rhodamine-conjugated anti-rabbit immunoglobulin G (1:200; Jackson Immunoresearch Laboratories, West Grove, PA) and DyLight 488-conjugated anti-mouse immunoglobulin G (1:1000; Rockland Immunochemicals Inc., Limerick, PA). After washing with PBS and staining with 4′,6-diamidino-2-phenylindole (Vector Laboratories, Burlingame, CA), the sections were preserved in VECTASHIELD mounting medium (Vector Laboratories) and examined and photographed with a fluorescence microscope Olympus BX-51 combined with Olympus DP72 camera and cellSens Standard 1.14 software (Olympus, Germany).

FIGURE LEGENDS

Figure S1 | Immunohistochemistry (IHC) analysis of Sirt1 expression in rat kidneys before and after unilateral ureteral obstruction (UUO). (**A**) IHC of Sirt1 in the sham-operated and obstructed kidney on days 7 after UUO (magnification, 40×). (**B**) Representative immunoblot and quantification of Sirt1 in the renal cortex and medulla in control and obstructed kidneys on days 7 after UUO are shown. IHC of Sirt1 in the cortex and medulla of kidneys of sham-operated rats (**C**), contralateral kidneys (**D**), and obstructed kidneys (**E**) on days 7 after UUO are shown (magnification, 200×). Sirt1 IHC intensity scores are shown for the above samples (**F**). #: $P = 0.003$, +: $P = 0.005$, *: $P < 0.001$ comparing with cortex.

Figure S2 | Immunohistochemistry (IHC) of Sirt1 in renal interstitium after UUO. IHC of Sirt1 in the renal interstitium of obstructed kidney on days 14 after UUO are shown (magnification, 400×). The arrows indicate increased Sirt1 expression in some interstitial cells.

Figure S3 | Immunofluorescence analysis of Sirt1, ED-1, and fibronectin in the kidney. In the kidneys of sham-operated rats (**A**) and the obstructed kidneys days 7 after UUO (**B**), immunofluorescence images of Sirt1 (red), ED-1 (green), and

4',6-diamidino-2-phenylindole (DAPI, blue) are shown. In the kidneys of sham-operated rats (**C**) and the obstructed kidneys days 7 after UUO (**D**), immunofluorescence images of Sirt1 (red), fibronectin (green), and DAPI (blue) are shown. The arrows indicate some of the co-localization of Sirt1 and ED-1 or fibronectin in the renal interstitium.

Figure S4 | Changes in the expression of angiotensin II type 2 receptor (AT2R) in the obstructed kidney 7 days after UUO and the effects of Sirt1 activation or inhibition. Representative immunoblot and quantification of AT2R in the kidneys of the controls, obstructed kidneys at 7 days after UUO (UUO), and obstructed kidneys with resveratrol (ResVe) or sirtinol (SirTi) intervention for 7 days after UUO.

Figure S1

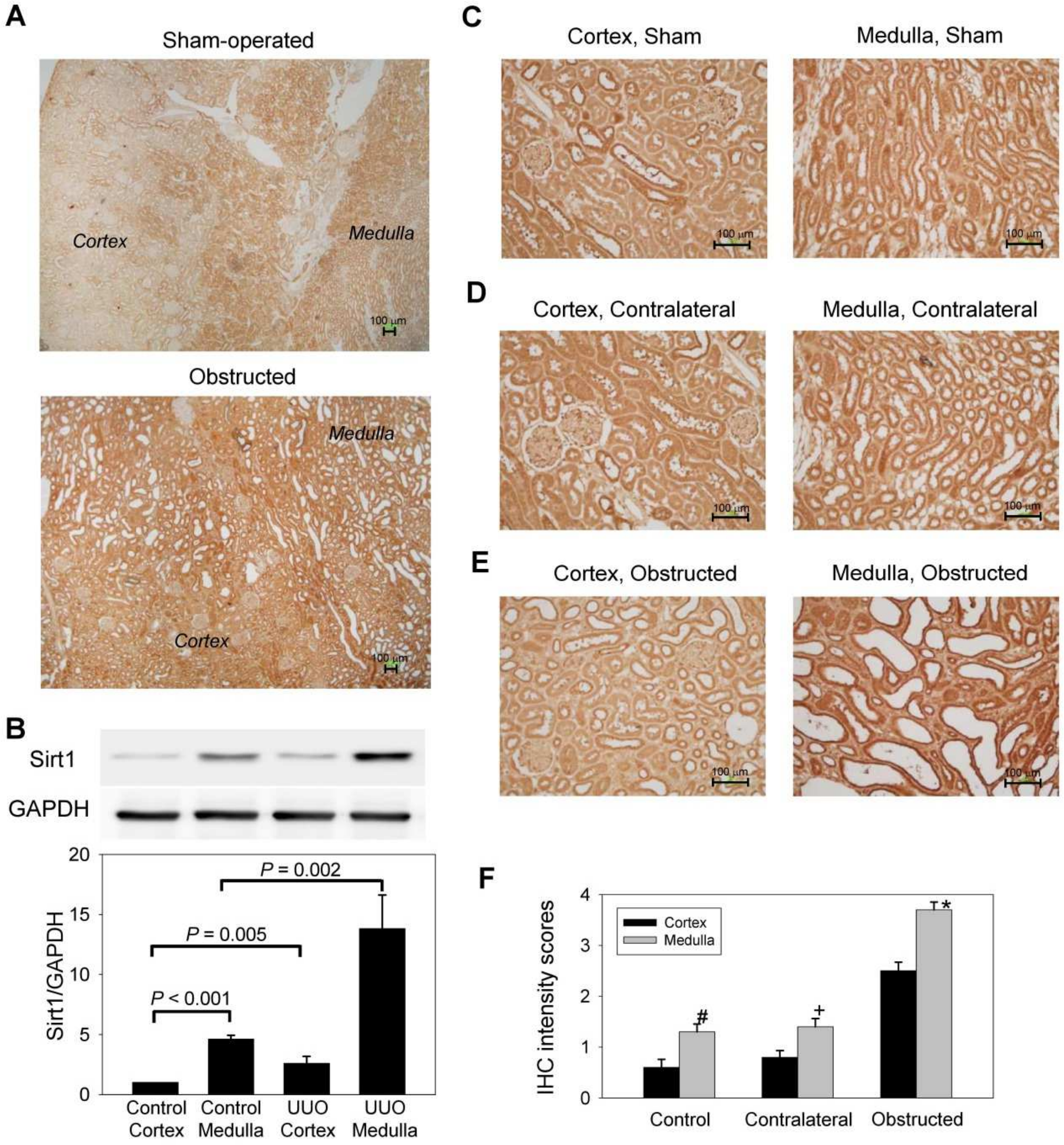
A
Sham-operated
Cortex
Medulla
100 µm
Obstructed
Medulla
Cortex
100 µm
B
Sirt1
GAPDH
20
15
10
5
0
Sirt1/GAPDH
P = 0.002
P = 0.005
P < 0.001
Control Cortex
Control Medulla
UUO Cortex
UUO Medulla
C
Cortex, Sham
Medulla, Sham
D
Cortex, Contralateral
Medulla, Contralateral
E
Cortex, Obstructed
Medulla, Obstructed
F
IHC intensity scores
Cortex
Medulla
#
+
*
Control
Contralateral
Obstructed

Figure S2

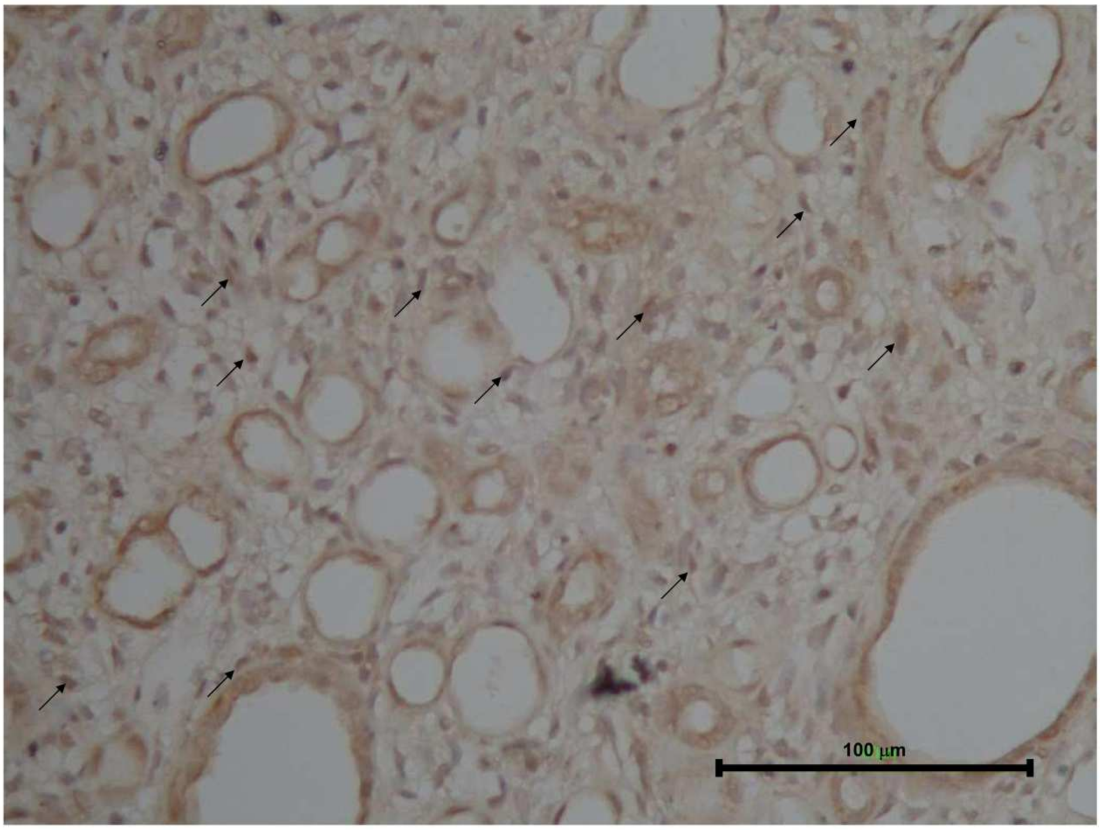

Figure S3

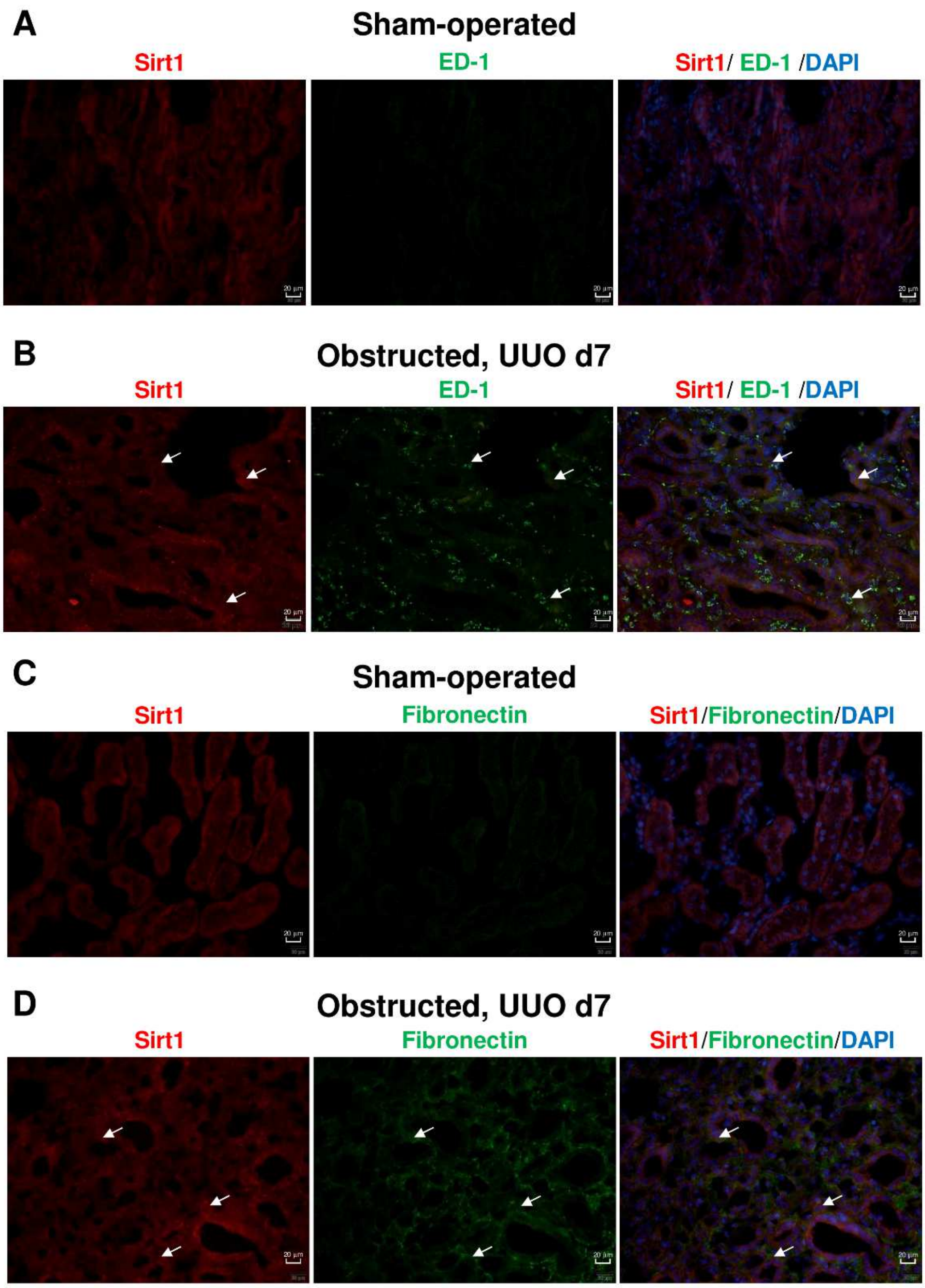
A
Sham-operated
Sirt1
ED-1
Sirt1/ ED-1 /DAPI
B
Obstructed, UUO d7
Sirt1
ED-1
Sirt1/ ED-1 /DAPI
C
Sham-operated
Sirt1
Fibronectin
Sirt1/Fibronectin/DAPI
D
Obstructed, UUO d7
Sirt1
Fibronectin
Sirt1/Fibronectin/DAPI

Figure S4

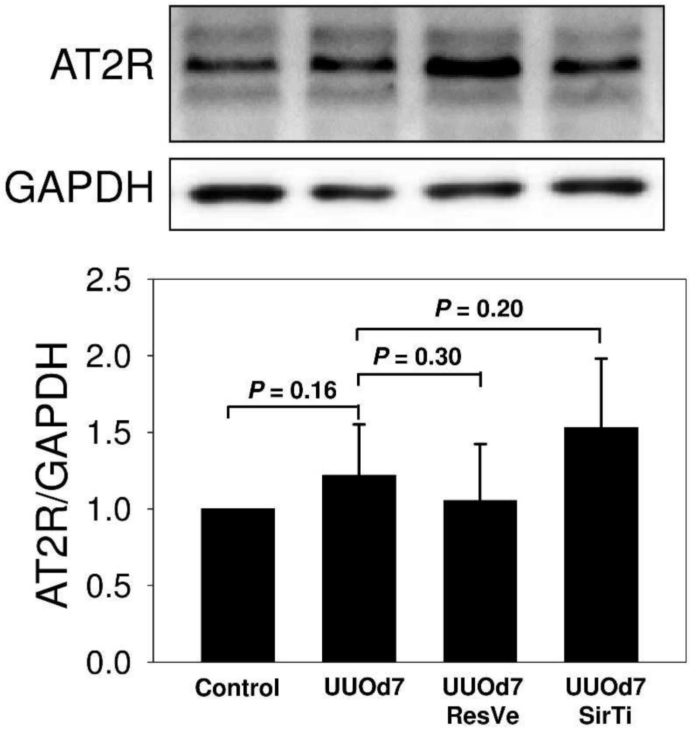
AT2R
GAPDH
2.5
2.0
1.5
1.0
0.5
0.0
AT2R/GAPDH
P = 0.20
P = 0.30
P = 0.16
Control
UUOd7
UUOd7
ResVe
UUOd7
SirTi
